# Increased extracellular release of microRNAs from dorsal root ganglion cells in a rat model of neuropathic pain caused by peripheral nerve injury

**DOI:** 10.1371/journal.pone.0280425

**Published:** 2023-01-20

**Authors:** Yuko Ikuma, Atsushi Sakai, Atsuhiro Sakamoto, Hidenori Suzuki

**Affiliations:** 1 Department of Anesthesiology, Nippon Medical School, Bunkyo-ku, Tokyo, Japan; 2 Department of Pharmacology, Nippon Medical School, Bunkyo-ku, Tokyo, Japan; Rutgers University, UNITED STATES

## Abstract

microRNAs (miRNAs) are extracellularly released by cells for intercellular communication, while intracellularly, they inhibit the expression of specific genes. An increasing number of studies suggest that extracellular miRNAs have great potential as both therapeutic targets and disease-specific biomarkers in a variety of diseases, including pain disorders. However, little is known about miRNA release from dorsal root ganglion (DRG) neurons in neuropathic pain caused by peripheral nerve injury. In this study, we investigated the changes in the extracellular release of miRNAs from DRG neurons in a rat model of neuropathic pain induced by chronic constriction injury of the sciatic nerve. We found increased release of six miRNAs (let-7d, miR-21, miR-142-3p, miR-146b, miR-203-3p and miR-221) from primary cultured DRG neurons prepared from rats 7 days after nerve injury. Among these, miR-221 was also increased in serum from days 7 to 28 after nerve injury. In contrast, serum miR-221 levels and its release from DRG neurons were unchanged in an inflammatory pain model produced by intraplantar injection of complete Freund’s adjuvant. These results suggest that the increased release of specific miRNAs by DRG neurons may be involved in the pathophysiology of neuropathic pain through extracellular as well as intracellular mechanisms. Furthermore, serum miR-221 may be useful as a biomarker of neuropathic pain caused by peripheral nerve injury.

## Introduction

Lesions or disease of the somatosensory system can cause neuropathic pain, which often has a chronic course [1]. Because of the limited effectiveness and severe adverse effects of current analgesics, neuropathic pain remains a major health problem worldwide [2, 3]. In addition, neuropathic pain can co-occur with other pain disorders, such as nociceptive pain caused by tissue inflammation. Therefore, it is often difficult to discriminate the neuropathic component in chronic pain when planning the treatment strategy. Thus, identifying novel minimally invasive biomarkers and elucidating the underlying pathophysiological processes are essential for developing effective therapies for peripheral neuropathies.

microRNAs (miRNAs) are small non-coding RNAs, typically ~22 nucleotides, that inhibit the expression of specific genes through translational repression or mRNA decay in a cell-autonomous manner [4]. miRNAs are also released from cells in extracellular vesicles (EVs) that can be taken up by other cells to function in a non-cell-autonomous manner [5]. Indeed, extracellular as well as intracellular miRNAs are involved in the pathophysiology of a variety of diseases, including pain disorders [6, 7]. For example, miR-21 upregulation in dorsal root ganglion (DRG) neurons contributes to neuropathic pain [8]. miR-21 released from DRG neurons is phagocytosed by macrophages to promote an inflammatory response [9]. Extracellular miRNAs are also involved in nociceptive pain induced by formalin injection [10] and in osteoarthritis pain [11], suggesting that they may have potential as therapeutic targets. Furthermore, miRNAs in liquid biopsy specimens can serve as minimally invasive biomarkers in a wide range of diseases, such as cancers, cardiovascular diseases and neurological disorders [7, 12]. Because miRNAs are encapsulated in EVs, they are stable in the extracellular space, and therefore can be detected in almost all body fluids, including blood, urine and cerebrospinal fluid. Extracellular miRNAs may therefore hold promise as both therapeutic targets and disease-specific biomarkers. However, although extracellular miRNAs have been reported to be released both constitutively and in a regulated manner [13], it remains unclear whether miRNA release from DRG neurons is affected by peripheral nerve injury, and if so, which miRNAs show a change in release.

In this study, we investigated the changes in the extracellular release of several miRNAs from DRG neurons after peripheral nerve injury. Furthermore, we examined whether the miRNAs showing enhanced release from DRG neurons were also increased in the serum, with the aim of identifying miRNAs that may serve as potential biomarkers for peripheral neuropathic pain.

## Materials and methods

### Animal models

All animal experiments were reviewed by the Animal Experiments Ethical Review Committee, were approved by the President of Nippon Medical School (Approval number 2020–042), and were carried out in accordance with the guidelines of the International Association for the Study of Pain [14]. Sprague-Dawley male rats (5 weeks old) were used for all experiments. All rats were housed in an animal room in a clean condition with free access to food and water. For surgery, all rats were deeply anesthetized with isoflurane inhalation (2–3%). Rats were sacrificed by exsanguination under deep anesthesia. For the neuropathic pain model, we used chronic constriction injury (CCI) [15]. CCI was produced by loose ligation of the left sciatic nerve with a 4–0 silk thread at 4 points. Nerve ligation was performed so that the toe on the fibula side twitched slightly. The sham model underwent the same procedure, but the nerve was not ligated. For the inflammatory pain model, complete Freund’s adjuvant (CFA; Merck KGaA, Darmstadt, Germany) was injected at a volume of 100 μl into the plantar surface of the left hind paw. In the control group, 100 μl of saline was injected into the left hind paw.

### Primary culture of DRG neurons

The lumbar fourth (L4) to sixth (L6) DRGs were dissected out and placed into a 30 mm dish containing 3 ml of Ham’s F12 nutrient mixture (Thermo Fisher Scientific, Waltham, MA, USA). The nerve fiber and membrane were carefully removed under a microscope on ice. The DRGs were cut into 4–8 pieces and incubated in 2.5 ml of PBS containing collagenase A (5 mg/ml; Roche Diagnostics, Basel, Switzerland) and dispase II (1 mg/ml; Roche Diagnostics) at 37°C for 30 min with shaking (80 rpm). Cells were centrifuged at 1,200 rpm for 1 min, and the supernatant was removed. The cells were further incubated with 3 ml of 0.05% Trypsin/0.53 mM EDTA·4Na solution (FUJIFILM Wako Pure Chemical Corporation, Osaka, Japan) at 37°C for 15 min with shaking (80 rpm). After centrifugation at 1,200 rpm for 1 min, the cell pellet was resuspended in 1 ml of Ham’s F12 nutrient mixture containing 15% fetal bovine serum. For neuronal enrichment, the cell suspension was overlayed onto 5 ml of 30% Percoll in PBS (GE Healthcare, Chicago, IL, USA) and centrifuged at 1,000 rpm for 5 min. The supernatant was removed and the cell pellet was washed twice with 6 ml of Ham’s F12 nutrient mixture. DRG neurons were suspended in 500 μl Neurobasal medium with 2% B27 supplement and 200 mM L-glutamine (Thermo Fisher Scientific). The DRG neurons were seeded onto poly-D-lysine/laminin-coated coverslips (Corning, Glendale, AZ, USA) and cultured in a humidified 5% CO_2_ incubator at 37°C. The medium was replaced the next day. Then, 24 h after medium replacement, the supernatant was collected for EV isolation.

### EV isolation

EVs were isolated from culture medium using the Total Exosome Isolation kit (from cell culture medium) (Thermo Fisher Scientific), according to the manufacturer’s instruction. Briefly, the culture medium was centrifuged at 2,000 × *g* for 30 min to remove cell debris. The Total Exosome Isolation reagent was added to the supernatant and incubated overnight at 4°C. The sample was centrifuged at 10,000 × *g* for 1 h at 4°C, and the pellet was collected as EVs. To isolate EVs from serum, blood was collected from rats by cardiac puncture. The blood was allowed to stand for 30 min at room temperature, and the serum was collected by centrifugation at 2,000 × *g* for 5 min. The serum was further centrifuged at 2,000 × *g* for 30 min to remove cell debris. Total Exosome Isolation (from serum) reagent (Thermo Fisher Scientific) was added to the serum and incubated for 30 min on ice. The sample was centrifuged at 10,000 × *g* for 10 min, and the pellet was collected as EVs.

### Quantitative reverse transcription-PCR

Total RNA was extracted from L4 DRGs and isolated EVs that were obtained from primary culture obtained from L4 to L6 DRGs using the RNAiso Plus kit (Takara Bio, Shiga, Japan) according to the manufacturer’s instruction. The concentration of the extracted RNAs was measured with the NanoDrop One Spectrophotometer (Thermo Fisher Scientific), and 10 ng of total RNA was used for reverse transcription. For EVs from culture medium, 1 μl of total RNA was used for reverse transcription because the RNA concentration was too low to quantify. Reverse transcription was performed using each miRNA-specific primer included in the TaqMan MicroRNA Assay (Thermo Fisher Scientific) and the TaqMan MicroRNA Reverse Transcription kit (Thermo Fisher Scientific). The reverse transcription conditions were 16°C for 30 min, 42°C for 30 min, and 85°C for 5 min (then held at 4°C). Quantitative PCR was performed using TaqMan Universal PCR Master Mix (Thermo Fisher Scientific) with TaqMan probe and a primer pair specific for the corresponding miRNA. The reaction was run on the StepOnePlus Real-Time PCR system (Thermo Fisher Scientific), with the following thermocycling profile: 95°C for 10 min, followed by 40 cycles of 95°C for 15 s and 60°C for 1 min.

### Behavioral test

The von Frey test was performed to assess paw withdrawal thresholds for mechanical stimuli before CCI surgery and on post-operative days 1, 4, 7, 14 and 28. Rats were placed on a metallic mesh floor covered with a plastic box, and a set of von Frey filaments (Muromachi Kikai, Tokyo, Japan) was applied to the hind paw from underneath. The trial consisted of application of a von Frey hair to the hind paw five times. The weakest force to which the rat responded at least three out of five stimuli was defined as the paw withdrawal threshold.

### Statistical analysis

Values are expressed as the mean ± SEM. SPSS software version 25 (IBM, Armonk, NY, USA) was used for statistical analyses. Normality of data was assessed with the Shapiro–Wilk test. If normal distribution was assumed, the unpaired *t*-test was performed for data sets. If the normal distribution was not assumed, the Wilcoxon signed-rank test or Mann–Whitney *U*-test was performed. Values of *P* <0.05 were considered statistically significant.

## Results

### Increase in extracellular miRNAs released from DRG cells after nerve injury

**Fig 1:**
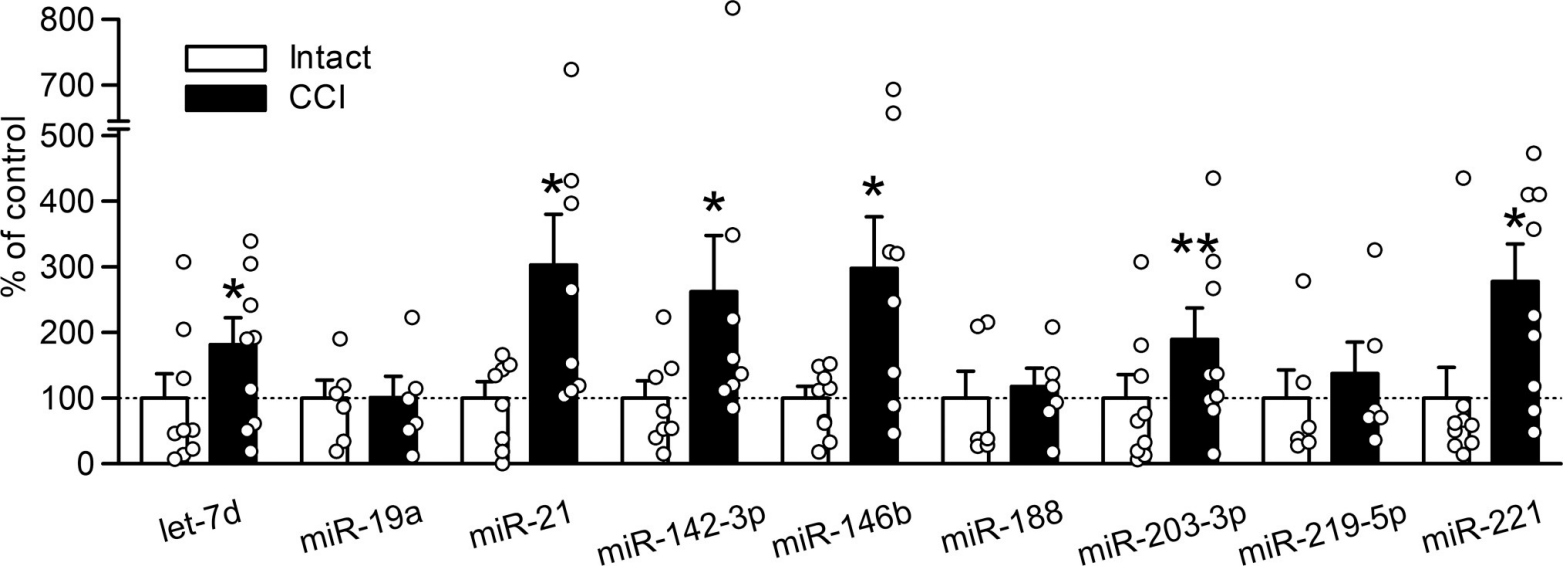
Extracellular miRNA levels in the culture medium of DRG cells after CCI. miRNA levels were examined in the EVs obtained from the culture medium of L4–L6 DRG neuron cultures on day 7 after CCI (*n* = 6–9). *P < 0.05 or **P < 0.01, compared with DRG neuron cultures obtained from the intact side (Wilcoxon signed-rank test).

To identify extracellular miRNAs with increased release from DRG cells after nerve injury, we focused on 9 miRNAs (let-7d, miR-19a, miR-21, miR-142-3p, miR-146b, miR-188, miR-203-3p, miR-219-5p and miR-221) that were previously reported to increase in the DRG after peripheral nerve injury [16–19]. Extracellular release of miRNAs was assessed using primary cultured DRG neurons prepared 7 days after CCI. Of the nine miRNAs tested, let-7d (*P* = 0.021), miR-21 (*P* = 0.012), miR-142-3p (*P* = 0.036), miR-146b (*P* = 0.021), miR-203-3p (*P* = 0.008) and miR-221 (*P* = 0.015) were significantly increased in the EVs released from the cultured DRG cells after CCI (Fig 1). On the other hand, secreted levels of miRNAs were not significantly changed by sham operation except for let-7d (S1 Fig). The observed increase in miR-21 release from DRG neurons was consistent with a previous report [9].

### Serum extracellular miR-221 is increased after CCI

**Fig 2:**
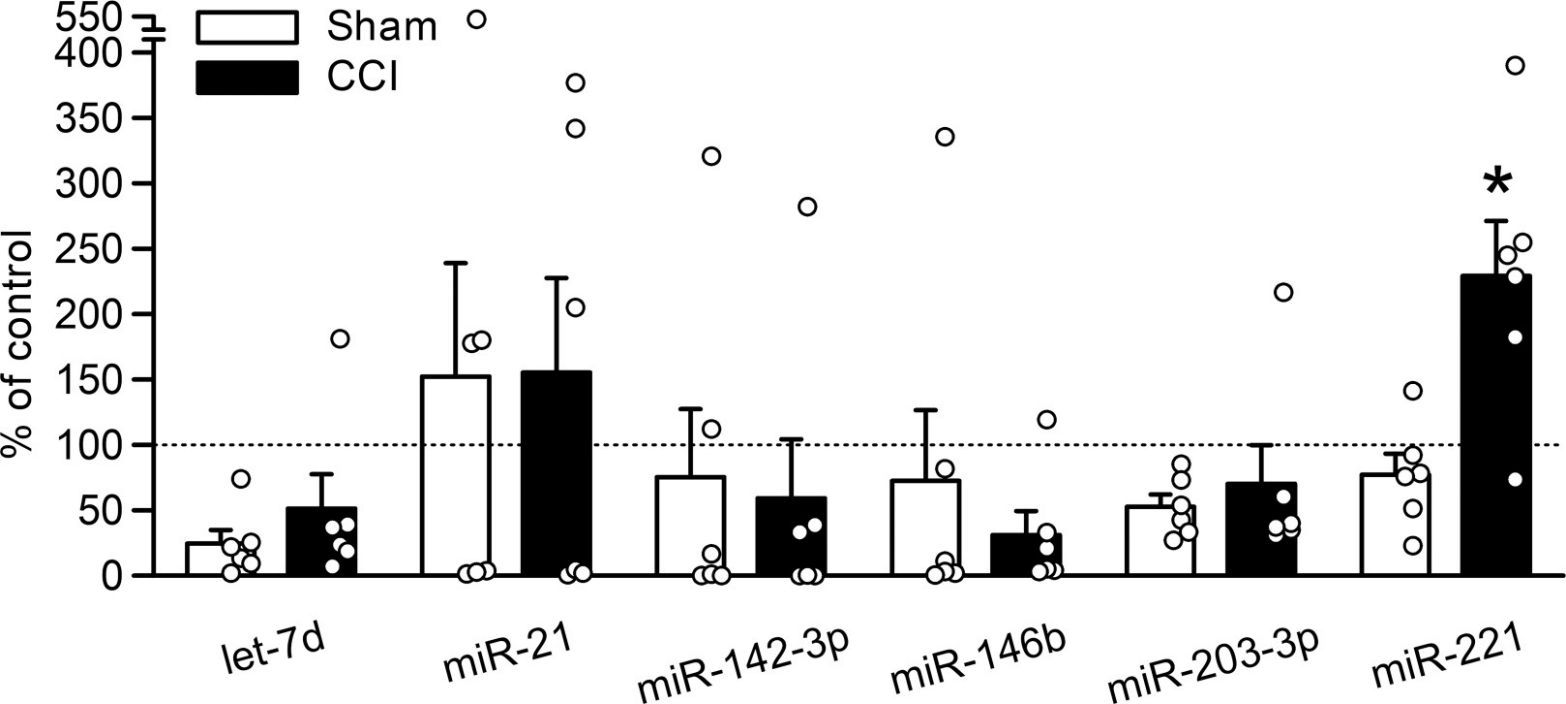
Serum levels of miRNAs after CCI. miRNA levels were examined in the EVs obtained from serum at day 7 after CCI (*n* = 6–9) and were shown as a percentage of the mean expression in naïve rats. *P < 0.05, compared with sham operation (Mann–Whitney *U*-test).

To investigate whether the increased release of miRNAs from DRG cells after nerve injury is reflected in changes in serum levels, serum extracellular miRNA levels were examined 7 days after CCI. Among the seven miRNAs that showed increased extracellular release from DRG cells, only miR-221 was significantly increased in the serum 7 days after CCI, compared with sham surgery (Fig 2; *P* = 0.025).

### Time-dependent increase in serum miR-221 levels after peripheral nerve injury

**Fig 3:**
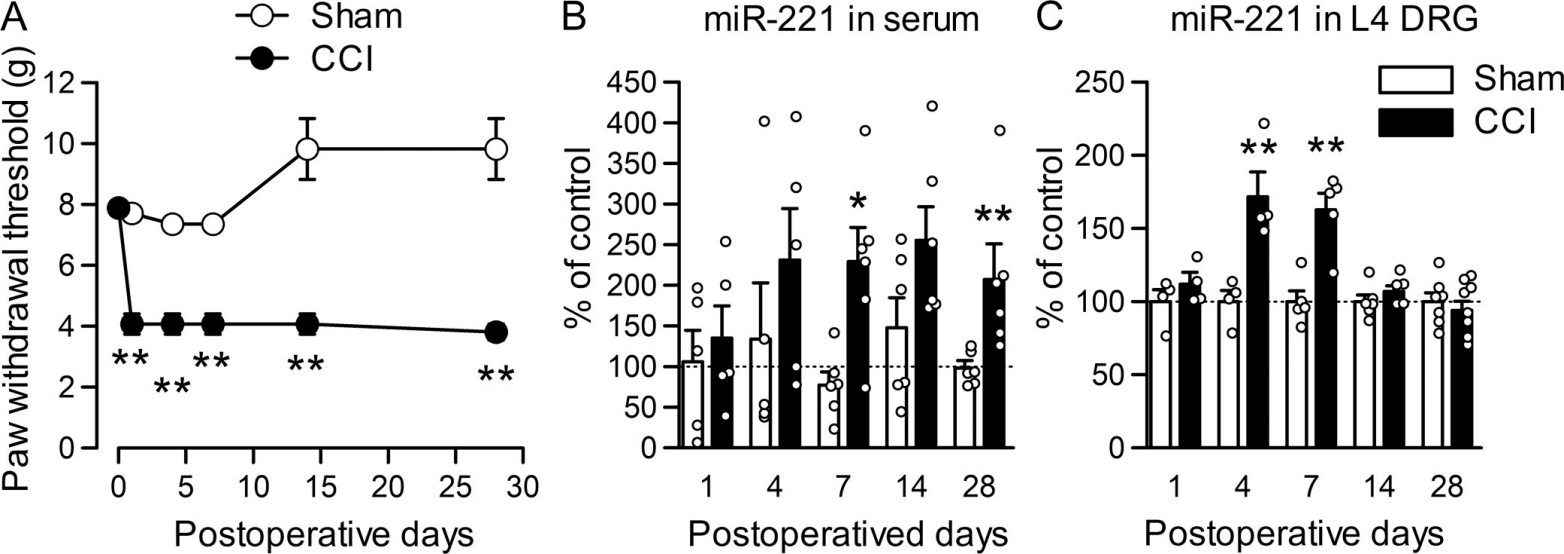
Time course of miR-221 changes in the serum and DRG after CCI. (A) Paw withdrawal responses to mechanical stimuli were evaluated before and after the CCI or sham surgery (*n* = 6). **P < 0.01, compared with sham operation (Mann–Whitney *U*-test). (B, C) miR-221 levels were examined in the EVs obtained from serum (B; *n* = 5–6) and L4 DRGs (C; *n* = 4–5) after CCI or sham surgery and were shown as a percentage of the mean expression in naïve rats. (B) Serum levels of miR-221 at day 7 after CCI (same as the data in Fig 2). *P < 0.05 or **P < 0.01, compared with sham operation (Mann–Whitney *U*-test) (B) and unpaired *t*-test (C).

Because serum miR-221 level was increased 7 days after peripheral nerve injury, we further investigated the time course of serum miR-221 change in rats with CCI. After CCI, withdrawal threshold to mechanical stimulus was significantly reduced at day 1, and the reduction persisted at least up to day 28 (Fig 3A; *P* = 0.003 for day 1, *P* = 0.002 for day 4, *P* = 0.002 for day 7, *P* = 0.003 for day 14 and *P* = 0.001 for day 28). Serum miR-221 was not significantly increased on days 1 or 4 after CCI compared with sham operation, but increased from day 7 to day 28 (Fig 3B; *P* = 0.025 for day 7 and *P* = 0.004 for day 28). In the ipsilateral L4 DRG, the miR-221 expression level began to increase from day 4 to day 7 after CCI, but returned to baseline level day 14 after CCI, compared with sham operation (Fig 3C; *P* = 0.008 for day 4 and *P* = 0.002 for day 7). miR-221 release was also enhanced in neighboring L1–L3 DRGs above injured DRGs (S2 Fig; *P* = 0.031).

### miR-221 level is unchanged after inflammation induced by CFA

**Fig 4:**
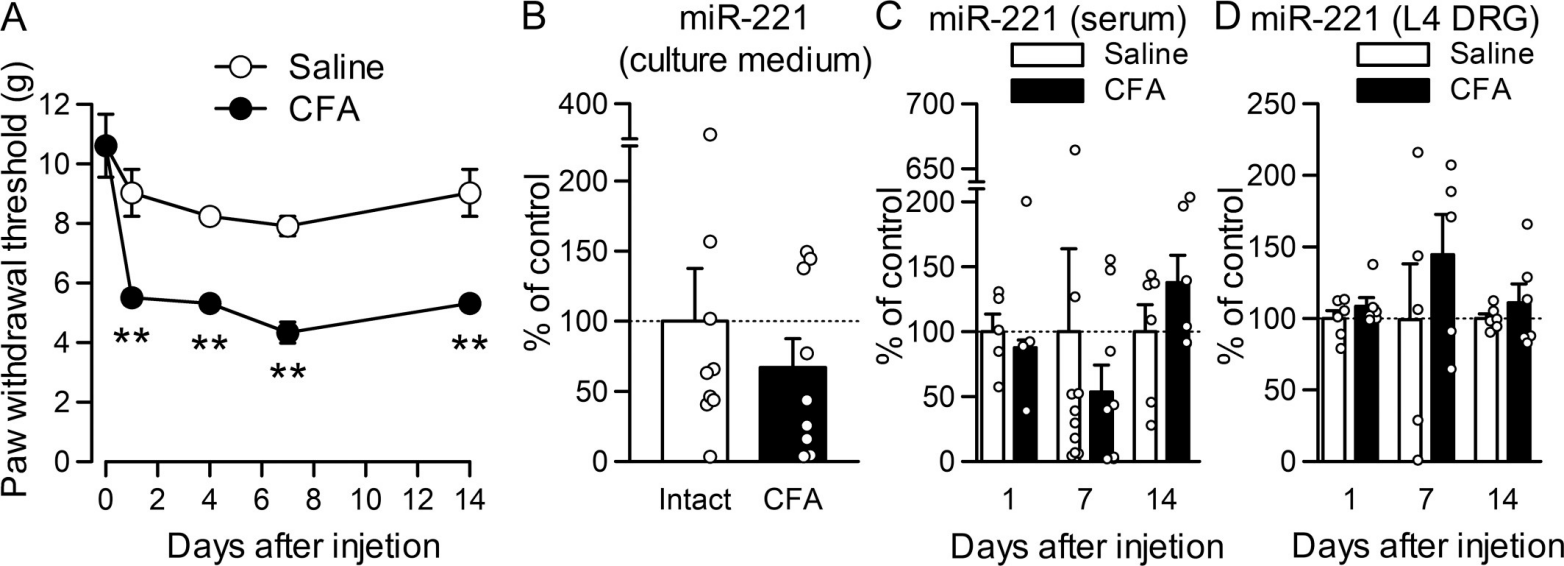
miR-221 levels after peripheral tissue inflammation induced by CFA. (A) Paw withdrawal responses to mechanical stimuli were evaluated before and after CFA or saline injection (*n* = 6). **P < 0.01, compared with saline injection (Mann–Whitney *U*-test). (B–D) miR-221 levels in the EVs obtained from culture medium obtained from L4-L6 DRGs on the injected side (B; *n* = 9) and serum (C; *n* = 9–10) and L4 DRG (D; *n* = 5).

miR-221 release was further examined in nociceptive pain caused by peripheral tissue inflammation, another major cause of pain. We induced inflammatory pain using CFA because it is a well-characterized inflammatory pain model. After CFA injection into the hindpaw foot pad, the withdrawal threshold to mechanical stimuli was significantly reduced from days 1 to 7 (Fig 4A; *P* = 0.002 for day 1, *P* = 0.001 for day 4, *P* = 0.002 for day 7 and *P* = 0.002 for day 14), as with CCI. However, the extracellular release of miR-221 was not significantly changed on day 7 after CFA injection, compared with DRG neuron cultures obtained from the intact side (Fig 4B). miR-221 levels in serum (Fig 4C) and L4 DRG that is a major constituent of the sciatic nerve (Fig 4D) were also unchanged from day 1 to day 14 after CFA injection compared with saline injection.

## Discussion

In the present study, we found that miRNAs are extracellularly released from DRG neurons in primary culture, and the release of some miRNAs was increased after peripheral nerve injury. In contrast, let-7d release was decreased by sham operation possibly due to the subtle injury or minor inflammation associated with sham operation. The miRNAs examined in the present study were reportedly upregulated in the DRG in several models of neuropathic pain [16–19]. However, not all of the miRNAs examined showed increased release after nerve injury. This suggests that the released miRNA does not simply reflect the amount of intracellular miRNA, and that the release of some EV-encapsulated miRNAs is regulated by subcellular mechanisms. Indeed, recent studies have identified several mechanisms by which specific miRNAs are selectively loaded into exosomes through RNA sequence motif and RNA-binding proteins [20–22]. In addition, miRNA release through EVs at least partly depends on neuronal activity [23]. In DRG neurons, the number of EVs and miRNA levels in the exosomal fraction are increased after capsaicin or high potassium chloride stimulation [9]. In this study, miR-221 level in serum was increased up to at least day 28, while its level in the DRG returned to the baseline. This might be due to the increased release from DRG or the release from other cells. Therefore, mechanisms underlying miRNA release by DRG neurons could be different among distinct miRNAs, as well as by the amount of miRNA expression and/or neuronal activity.

There is accumulating evidence that intracellular miRNAs in DRG neurons play key roles in development and/or the maintenance of neuropathic pain [24, 25]. In addition to acting on the cells producing them, some miRNAs were reported to function as intercellular (paracrine) signaling molecules in the pathophysiology of neuropathic pain by acting on other neuronal and/or non-neuronal cells. For example, let-7b is released from DRG neurons by nociceptive stimuli and elicits rapid spontaneous pain via TRPA1 activation through TLR7 [10]. In addition, miR-21 released from DRG neurons is taken up by neighboring macrophages and promotes a pro-inflammatory phenotype in neuropathic pain [9]. Furthermore, extracellular miR-21 activates the TLR7 receptor in DRG neurons, contributing to pain in osteoarthritis [11]. Collectively, these findings suggest that the increase in miRNA release from DRG cells observed in this study may also work as a pain mediator. Regarding the miR-221, its enhanced release may be potentially involved in maintenance rather than initiation of neuropathic pain because miR-221 in serum EVs was elevated 7 days after injury but not early after injury when the neuropathic pain had already established. The functional significance of intracellular and extracellular miRNAs needs to be investigated to fully understand the pathophysiological involvement of miRNAs in neuropathic pain.

The increased expression of miR-221 in DRG neurons has been reported in different types of peripheral nerve injury, including spinal nerve ligation [16, 18], sciatic nerve resection [19] and dorsal and ventral root transection [16]. Intravenous injection of lentiviral vector encoding miR-221 inhibitor reduces pain in rats with diabetic peripheral neuropathy [26], although the site of action remains unclear. CCI increases miR-221 expression in microglia in the spinal dorsal horn, and the intrathecal injection of miR-221 inhibitor alleviates neuropathic pain by reducing proinflammatory cytokine expression through targeting suppressor of cytokine signaling 1 (SOCS1) [27]. Because miR-221 was also increased in serum, circulating miR-221 may also affect other DRGs to produce hyperalgesia. Therefore, it is intriguing to investigate whether miR-221 is released from DRG neurons into the spinal cord and activates microglia.

The source of the increased serum miR-221 remains unclear. Accumulating evidence shows that EVs containing miRNAs and proteins can cross the blood–brain barrier in both directions [28, 29]. Given that miR-221 can enter the vascular system from sequestrated parts of the nervous system, such as DRGs and the spinal cord, the increase in miR-221 in serum EVs might reflect an increase in release from DRG cells. However, because spinal microglial cells increase miR-221 after CCI [27], a contribution of spinal miR-221 cannot be excluded. Interestingly, L1–L3 DRG cells adjacent levels above injured L4–L6 DRGs also increased miR-221 release. Although the underlying mechanism by which the increased release from injury-spared adjacent DRG cells was unknown, extracellular messengers, such as miR-221 itself or cytokines, may be involved. A way to identify the source of miRNAs is to characterize EVs that contain the miRNAs of interest. By analyzing the tissue-specific markers of EVs, it may be possible to collect origin-specific EVs and measure miRNAs contained only in the EVs of interest. Indeed, a subpopulation of EVs of neuronal origin can be obtained by immunoprecipitating EVs with neuronal surface markers such as NCAM and/or L1CAM [29].

Although its cellular origin is currently unknown, miR-221 may be useful as a biomarker of neuropathic pain states, because its serum level was specifically increased in association with increased release from DRG cells. Furthermore, in contrast to CCI, increase in serum miR-221 was not observed after CFA, a well-characterized inflammatory pain model. Given that pain is a subjective experience and is difficult to measure with biochemical indices, objective biomarkers would be very clinically useful for assessing the presence/extent of peripheral nerve injury and in determining the primary cause of pain states. Consistent with the present study, Wu et al. [26] reported an increase in miR-221 levels in serum exosomes in a rat model of diabetic peripheral neuropathy. In comparison, Xu et al. reported a decrease in serum miR-221 in rats with spinal nerve ligation [30], although expression in the DRG is upregulated after CCI (in this study) as well as spinal nerve ligation [16, 18]. The discrepancy may result from differences in sample preparation. EVs were extracted from serum in the present study for the miRNA analysis, while whole serum was used by Xu et al. [30]. In addition to miR-221, several miRNAs have been reported to undergo changes in expression in liquid biopsy samples, including blood, in patients with pain disorders [25]. Given the difference in serum miR-221 change between CCI and CFA treatments, miR-221 could be useful as a biomarker of neuropathic pain caused by peripheral nerve injury.

## Supporting information

S1 Fig miRNA levels in the culture medium after sham operation. miRNA levels were examined in the EVs obtained from the culture medium of L4–L6 DRG neuron cultures obtained from naïve rats and sham-operated rats on day 7 (*n* = 6). *P < 0.05, compared with naïve rats (Mann–Whitney *U*-test).
(PDF)

S2 Fig miR-221 level in the culture medium of L1–L3 DRGs after CCI. miR-221 level was examined in the EVs obtained from the culture medium of L1–L3 DRG neuron cultures on day 7 after CCI (*n* = 6). *P < 0.05, compared with DRG neuron cultures obtained from the intact side (Wilcoxon signed-rank test).
(PDF)
