# Supplementary material for: Increased extracellular release of microRNAs from dorsal root ganglion cells in a rat model of neuropathic pain caused by peripheral nerve injury
Source: PLoS One. 2023 Jan 20;18(1):e0280425. doi: 10.1371/journal.pone.0280425 (PMC9858844; doi:10.1371/journal.pone.0280425)
Supplement: S1 Fig — miRNA levels were examined in the EVs obtained from the culture medium of L4–L6 DRG neuron cultures obtained from naïve rats and sham-operated rats on day 7 (n = 6). *P < 0.05, compared with naïve rats (Mann–Whitney U-test). (PDF) [file pone.0280425.s001.pdf]

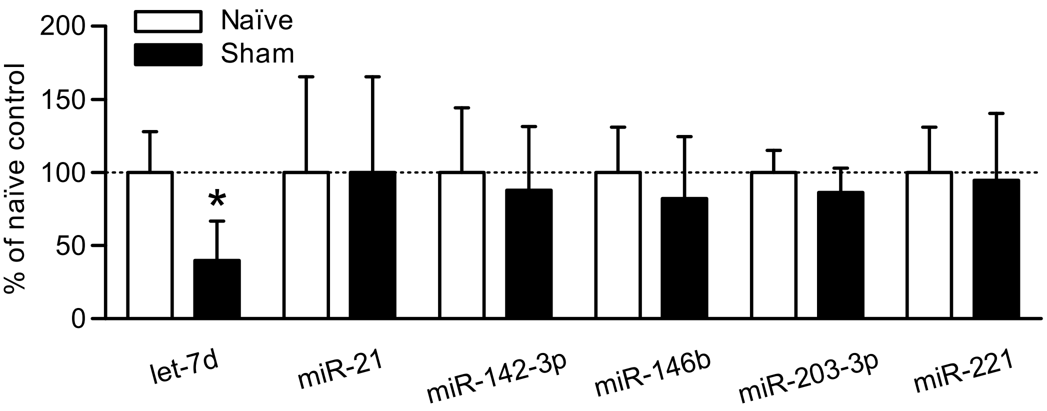

S1 Fig. miRNA levels in the culture medium after sham operation.

miRNA levels were examined in the EVs obtained from the culture medium of L4–L6 DRG neuron cultures obtained from naïve rats and sham-operated rats on day 7 ($n = 6$). $^*P < 0.05$, compared with naïve rats (Mann–Whitney *U*-test).
