# Supplementary material for: Increased extracellular release of microRNAs from dorsal root ganglion cells in a rat model of neuropathic pain caused by peripheral nerve injury
Source: PLoS One. 2023 Jan 20;18(1):e0280425. doi: 10.1371/journal.pone.0280425 (PMC9858844; doi:10.1371/journal.pone.0280425)
Supplement: S2 Fig — miR-221 level was examined in the EVs obtained from the culture medium of L1–L3 DRG neuron cultures on day 7 after CCI (n = 6). *P < 0.05, compared with DRG neuron cultures obtained from the intact side (Wilcoxon signed-rank test). (PDF) [file pone.0280425.s002.pdf]

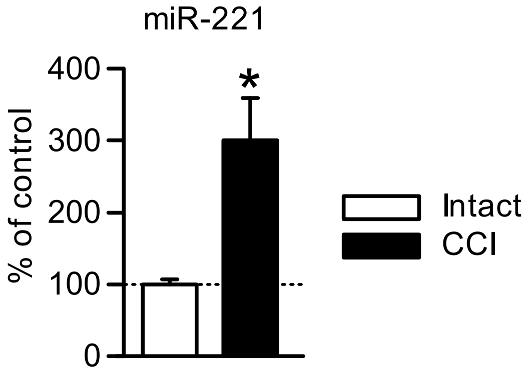

S2 Fig. miR-221 level in the culture medium of L1–L3 DRGs after CCI.

miRNA levels were examined in the EVs obtained from the culture medium of L1–L3 DRG neuron cultures on day 7 after CCI (n = 6). *$P < 0.05$, compared with DRG neuron cultures obtained from the intact side (Wilcoxon signed-rank test).
